# Rise and dine: unraveling breakfast habits among tenth graders - a cross-sectional study among 646 students in the City of Witten, Germany (GeWIT study)

**DOI:** 10.1186/s12889-025-23002-w

**Published:** 2025-05-15

**Authors:** Mohamed Salah Anwar Mohamed, Nessia Rachma Dianti, Anne-Lisa Heye, Klaus Völkel, Judith Tillmann, Oxana Klassen, Paul Wiesheu, Klaus Weckbecker, Eva Münster

**Affiliations:** 1 Institute for General Medicine and Primary Care, Chair of General Medicine I and Interprofessional Care, University of Witten/Herdecke, Alfred-Herrhausen-Street 50, 58455 Witten, Germany; 2 Institute for General Medicine and Primary Care, Chair of General Medicine II and Patient-Centredness in Primary Care, University of Witten/Herdecke, Witten, Germany; 3 Office for Labor, Health Economy, Technology Transfer, and University Development, City of Witten, Witten, Germany

**Keywords:** Adolescents, Breakfast skipping, Health behaviours, Malnutrition, Healthcare, Dietary patterns, Sociodemographics

## Abstract

**Background:**

Despite widespread recognition of breakfast as a key contributor to adolescent health, breakfast skipping remains common and concerning. It has been associated with increased consumption of energy-dense snacks, lower intake of essential nutrients, and irregular eating patterns throughout the day. This study aimed to assess the prevalence of breakfast skipping on the day of the survey among adolescents in Witten, Germany, and to explore its associations with selected sociodemographic characteristics, health-related behaviours, and self-reported health indicators.

**Methods:**

Between November 2, 2021, and February 25, 2022, a cross-sectional, survey-based study was conducted among 10th-grade students in all nine municipally managed secondary schools in Witten. The questionnaire assessed breakfast consumption on the surveyed day, school type, sociodemographic factors, health status, and physical activity. Statistical analyses were conducted using SPSS, including chi-square tests and multivariable logistic regression to estimate odds ratios and identify predictors of breakfast skipping.

**Results:**

In a sample of 646 students (response rate 98.3%), 50.6% (*n* = 327) reported skipping breakfast before school on the day of the survey. Students attending intermediate secondary schools (aOR 1.66, 95% CI 1.1–2.51) or comprehensive schools (aOR 1.62, 95% CI 1.11–2.36), and those with a migration background (defined in Germany as having at least one parent born abroad) (aOR 1.46, 95% CI 1.04–2.04), were significantly more likely to skip breakfast. In contrast, students reporting “excellent” (aOR 0.29, 95% CI 0.17–0.48) or “very good” (aOR 0.63, 95% CI 0.43–0.91) general health, and those categorized as underweight (aOR 0.44, 95% CI 0.2–0.98), were significantly less likely to skip breakfast. No significant associations were found for gender, age, socioeconomic status, physical activity, or family doctor consultations.

**Conclusion:**

This study indicates that the educational environment and cultural background might significantly impact adolescents’ breakfast consumption behaviours on the surveyed day, underscoring the need for targeted educational interventions. Promoting a positive perception of personal health is key to encouraging regular breakfast consumption among adolescents. These findings provide important insights for public health strategies to enhance adolescents’ dietary habits.

**Trial registration:**

Not applicable.

**Supplementary Information:**

The online version contains supplementary material available at 10.1186/s12889-025-23002-w.

## Background

Food is the primary external source of energy and essential nutrients required for physiological functions and overall health [1]. Research continuously reveals an increasing number of food compounds associated with health benefits [2]. It is essential to understand the connections between specific food groups, their nutrient compositions, and overall dietary patterns, especially concerning the prevention and progression of chronic diseases, including cardiovascular diseases, various cancers, chronic respiratory conditions, and diabetes [1, 3, 4]. Furthermore, adopting nutritionally balanced eating habits can improve overall health and reduce the risk of diet-related diseases [5–7].

Numerous studies have indicated that maintaining a consistent meal routine, especially consuming breakfast, can improve overall diet quality and mental health: for instance, a study conducted in the United States by Rampersaud et al. (2005) summarizes the results of 47 studies examining the association of breakfast consumption with nutritional adequacy, body weight, and academic performance in children and adolescents. These studies, primarily conducted in various populations in the United States and Europe, found significant improvements in diet quality and mental health associated with regular breakfast consumption. This underscores the notion that breakfast as part of a healthful diet and lifestyle can positively impact children’s health and well-being [8]. Another study by Van Lippevelde et al. (2012) in Europe examined children aged 10 to 12 years and highlighted the positive associations between regular breakfast consumption, BMI, overall dietary quality, nutritional profiles and improved cognitive performance in a diverse sample across several European countries [9]. Additionally, research by Baldinger et al. (2012) with a total of 656 schoolchildren, aged 7 to 10 years in Switzerland explored the impact of regular breakfast consumption on BMI, motor functional skills and mental health among children, further supporting the benefits of regular breakfast intake [10]. Throughout the night, the body utilizes stored energy, which is depleted by morning; breakfast serves to replenish this energy [11]. Individuals who skip breakfast tend to have lower intakes of several essential nutrients, including vitamins A, E, C, B6, B12, folate, iron, calcium, phosphorus, magnesium, and dietary fiber, compared to those who eat breakfast. These nutritional shortfalls are rarely compensated for in subsequent meals [11]. Breakfast is frequently described as the day’s most crucial meal contributing significantly to the nutrient adequacy of the whole diet [8]. Breakfast consumers are more likely to have better overall diet quality, and micronutrient, macronutrient and fibre intakes that more often align with current dietary recommendations [8, 12, 13]. Moreover, breakfast consumption enhances cognitive function particularly memory and academic performance, as well as psychosocial functioning and school attendance among children and adolescents [8–10]. It helps alleviate hunger, which is linked to emotional and academic problems [14]. These benefits may stem from both immediate metabolic effects and the long-term provision of essential nutrients that support healthy brain development and function [15]. Two randomized controlled trials demonstrated that providing a school breakfast had a positive impact on nutritional status, test scores and school attendance rates among children in rural Jamaica [8]. In these studies, groups of children were monitored for two semesters (*n* = 115) [14] or for a year (*n* = 814) [16] during the intervention period and showed better performance compared to the control group, who did not receive a school breakfast. Additionally, a trial involving Peruvian children, who were randomly assigned to either receive or not receive a school breakfast over a period of three months, reported improved school attendance rates among those who received the breakfast [17]. However, the interpretation of existing data on breakfast consumption habits and related health outcomes may be influenced by factors such as socioeconomic status (SES) or other social and educational variables [18–21].

Adolescence is a crucial period characterized by rapid physical and psychological transformations, presenting an ideal opportunity to establish health behaviours that impact long-term well-being [7, 22]. Additionally, Dietary habits and taste preferences developed during childhood and adolescence are likely to continue into adulthood and can even last for a lifetime [4, 6, 7]. Unlike children, whose cognitive abilities are still developing, adolescents have the maturity to consciously evaluate their food choices and eating behaviours [5, 7]. At this stage, family influence on eating patterns decreases as individual responsibility and independence grow. Consequently, this phase presents an optimal opportunity for effective nutritional education and dissemination of information [5].

Studies in Switzerland and several European countries have shown that children and adolescents who regularly eat breakfast tend to have a lower Body Mass Index (BMI) and are less likely to be overweight [9, 10]. Consuming breakfast may help in reducing the likelihood of snacking on high-calorie foods [8]. Skipping breakfast is a common behaviour observed in overweight or obese children and adolescents in European studies and may be related to dieting and disordered eating habits [18, 23]. Breakfast skippers may be less likely to engage in physical activity, which may contribute to positive energy balance and weight gain [9, 10, 19, 24].

Although most previous studies have highlighted a link between skipping breakfast and obesity, other research has also indicated a correlation between breakfast skipping and malnutrition and being underweight. For instance, a 2005 cross-sectional study in urban India with 802 schoolchildren found a significant correlation between breakfast skipping and being underweight, with 53.8% of daily breakfast skippers being underweight versus 9.9% of non-skippers. Breakfast skippers had lower intakes of energy and protein, resulting in a daily caloric deficit of 600–900 kcal, highlighting the importance of regular breakfast for better nutritional status and healthier weight in children [25]. While these findings reflect the nutritional challenges in those regions, we acknowledge that such contexts differ considerably from Germany in terms of socioeconomic structures, cultural food practices, and overall diet quality. Therefore, caution is warranted when extrapolating these associations to a high-income country setting. Nonetheless, these studies underscore the importance of breakfast as a protective factor against inadequate nutritional intake.

Moreover, the collected data from various studies consistently show that breakfast consumption is closely linked to healthier weight status, with breakfast consumers being less likely to be underweight than those who skip breakfast [26]. Furthermore, the Diet Diversity Score analysis (DDS) [27] across different age groups highlighted a significant association between breakfast consumption and higher food diversity. For example, a study conducted in Japan last year found that underweight women, especially younger ones, tended to skip breakfast and had less variety in their diets [28]. Another study conducted as part of the 2011–12 National Nutrition and Physical Activity Survey aimed to assess the prevalence and factors associated with skipping breakfast among 1,592 Australian children and adolescents aged 2 to 17 years. Skipping breakfast was found to be more common among girls, older children, and adolescents. Adolescents were 4.1 times more likely to skip breakfast compared to younger children. Both a higher BMI and being underweight were linked to a greater likelihood of skipping breakfast [29].

Beyond its nutritional value, food carries important social and cultural meaning, especially during adolescence. Breakfast habits are often shaped by family routines, cultural norms, and social dynamics such as commensality; the practice of eating together [30]. Shared meals offer more than nutritional value, they help structure daily routines and foster meaningful family interactions [31]. They provide a space for discussing concerns and positive experiences, strengthening emotional bonds and parental involvement in children’s lives [30].

Despite the substantial benefits of consuming breakfast, the trend of skipping breakfast has risen among children and adolescents worldwide over recent years and the frequency of regular breakfast intake appears to decline with age [6, 26, 32, 33].

Breakfast skipping among adolescents is influenced by a range of psychosocial and structural factors. The HBSC study found that gender is a significant determinant, with girls more likely than boys to skip breakfast regularly, potentially reflecting weight control motives and body image concerns. In contrast, boys were more likely to skip breakfast due to practical reasons such as lack of time or low appetite in the morning. The study also highlighted the role of socioeconomic status, showing that adolescents from families with lower socioeconomic backgrounds were less likely to eat breakfast daily [34].

While many behavioural and psychosocial determinants of breakfast skipping such as body image concerns, dieting behaviours, and family routines have been well documented in the literature, our study did not directly assess these factors. Instead, the focus was on quantifiable sociodemographic and health-related variables, including school type, migration background, BMI, subjective health status, and physical activity. There remains a significant gap in the literature regarding the specific factors influencing breakfast consumption behaviours among adolescents, particularly across diverse socioeconomic and cultural contexts in Germany.

Therefore, examining the associations between breakfast consumption and both school type and migration background is essential for understanding dietary disparities and informing targeted interventions. Our study contributes to filling this gap by conducting a survey among adolescents in Witten, Germany, on this topic. Understanding potential associations is essential for informing targeted interventions aimed at promoting healthy eating behaviours from an early age.

## Methods

### Aim of the study

This study investigates breakfast skipping on the day of the survey among adolescents in tenth grade in Witten, Germany, and examines its association with a range of sociodemographic, lifestyle, and health-related factors. While previous research has explored the relationship between breakfast habits and health behaviours, evidence from Germany (particularly among 10th-grade students) remains limited. By analysing data from a representative sample, this study addresses key gaps in the literature and offers several novel insights that may be relevant to the broader German adolescent population. Notably, it identifies school type as an independent predictor of breakfast skipping, a factor that has received little attention compared to socioeconomic status or parental influence. The study also highlights that students with a migration background are significantly more likely to skip breakfast, pointing to the importance of considering cultural and structural influences. Additionally, self-rated health emerges as a strong predictor of breakfast consumption, offering a new perspective beyond the commonly studied link with BMI. Interestingly, the findings reveal that underweight adolescents are significantly less likely to skip breakfast, contrasting with prior studies that primarily associate breakfast skipping with overweight or obesity. These insights contribute valuable evidence to support the development of targeted public health strategies aimed at improving adolescent dietary habits in Germany.

### Study design and setting

From November 2, 2021, to February 25, 2022, a questionnaire-based cross-sectional study was conducted among students in nine out of nine secondary schools under municipal management in Witten, Germany. The study was carried out in the classrooms of the nine schools across 28 classes during school hours, in consultation with the school administrations, to maximize student participation. The study was conducted as part of the “Healthy City Witten” (GeWIT) project funded by the statutory health insurance “Techniker Krankenkasse”.

### Study population and recruitment, execution

The survey targeted all 10th-grade students from secondary schools in Witten, a city in North Rhine-Westphalia, Ennepe-Ruhr District, Germany, situated in the Ruhr metropolitan region and home to approximately 96,000 residents [35]. Witten has a total of 32 educational institutions, including 18 primary schools, 9 secondary schools, 4 special and alternative schools and the University of Witten/ Herdecke.

At the time of the survey, students who were present in class participated, achieving a high response rate of 98.3%. Exclusions from the study included two Waldorf schools, which did not support the study, and two special education schools, requiring a different survey approach. The final analysis incorporated data from 646 students, after excluding those not present (*n* = 95), underage (< 15 years old, *n* = 2), those who declined participation (*n* = 7), provided joke responses (*n* = 4), or had missing data on having breakfast (*n* = 3).

Following approval by the Ethics Committee, the project was carried out in nine municipal secondary schools in Witten, Germany. The survey instrument, including the study protocol, was formally approved by the Ethics Committee in an amendment dated July 29, 2021. Before data collection started, authorization was sought by contacting the school authority of the Ennepe-Ruhr District. Subsequently, the school administrators were contacted in writing by employees of the institute conducting the study and informed about the content and purpose of the study. After permission was granted, the preparatory steps for implementation were undertaken. Two weeks before the study implementation, two separate information letters for students and parents/guardians were sent to school administrators or designated contact persons from the teaching staff, which were then forwarded to students and parents/guardians. Participating teachers were informed by the school administrators. On regular school days, data were collected in 28 classes by means of self-administered questionnaires, which students completed during lesson time in agreement with school administrators. This is intended to reach a broad coverage of the students. Prior to completion, participants were informed about the aims of the project, inclusion and exclusion criteria, voluntary participation, data protection, anonymity, and the option to withdraw at any time. Filling in and submitting the questionnaire, which required around 25 minutes, was considered to represent consent. Ethical approval and clearance regarding data protection were obtained from the Ethics Committee of Witten/Herdecke University (ethics approval no. 97/2019; data protection votes no. DT-537 and DT-630). The study received financial support from the statutory health insurance provider Techniker Krankenkasse.

### Survey instrument

A 13-page, standardized questionnaire to be completed in writing by the students was used as the survey instrument (for this paper, the questionnaire was uploaded as a separate supplementary file both in the original German language and translated into English [36]).

It included questions on health and physical activity, emotions, family, friends and leisure, school situation, bullying, nutrition, physical development, health behaviour, competence, and promotion, social media, use of medical services, the COVID-19 pandemic, and sociodemographics. The responses were given through single-answer fields, partly in the form of Likert scales, multiple-answer options, and additional free-text fields.

For our paper, the following variables were analysed:

- Did you have breakfast before going to school today (on the day of the survey)? (yes or no).
- Sociodemographics: Gender (girls, boys or diverse), age (15,16 or 17–19), school type (academic high school, intermediate secondary school, comprehensive school, or general secondary school), subjective socioeconomic status (high, moderate, or low) and migration background (yes or no).
- Health related parameters: Frequency of weekly physical activities (3 or more days weekly, 1–2 days weekly or no weekly physical activities), BMI (underweight, normal weight, overweight or obese), family doctor’s consultations last year (0–2 times yearly, 3–5 times yearly, 6 or more times yearly) and subjective health status (excellent, very good, good, or less good/bad).

The questionnaire used in our study was developed based on validated instruments from the German Health Interview and Examination Survey for Children and Adolescents (KiGGS), conducted by the Robert Koch Institute, and the internationally validated Health Behaviour in School-aged Children (HBSC) study coordinated by the WHO. These instruments have been widely applied in national and international adolescent health research and are known for their methodological robustness [37, 38]. To ensure clarity and age-appropriateness, a pretest was conducted with five 10th-grade students prior to the full survey implementation. Based on their feedback, minor linguistic adjustments were made to improve comprehension, and a few additional questions were added.

### Statistical analyses

The data were transferred into digital form using the Scan-System FormPro. They were then checked for plausibility, processed, and analysed using the statistical evaluation program IBM SPSS Statistics, Version 28, through uni-, bi-, and multivariate analyses. An error probability of α = 0.05 was assumed. Missing data were noted as a separate category in the descriptive analysis if they accounted for 5% or more of cases. Potential differences regarding sociodemographic and health information were calculated using Chi-square tests.

The variable “migration background” was calculated based on the definition provided by the German Federal Statistical Office [39]. According to the Federal Statistical Office of Germany (DESTATIS), an individual is considered to have a migration background if they, or at least one of their parents, were not born with German citizenship. This includes both individuals who have immigrated to Germany and those born in Germany with at least one parent who immigrated or was born as a foreigner in Germany. BMI was calculated from self-reported height and weight using the Kromeyer-Hauschild criteria [40]. SES (subjective socioeconomic status) was calculated using the McArthur Scale, which is also used in the KIGGS study by the Robert Koch Institute (RKI, the official public health institute of Germany) [41].

Binary logistic regression analyses were conducted with having breakfast and skipping breakfast before school on the day of the survey as the dependent variable. The previously discussed sociodemographic or health related variables were included as independent variables. Adjusted odds ratios (aOR) with 95% confidence intervals were calculated after adjusting for age, gender, and the other additional parameters. Missings in the independent variables in the logistic regression model were assigned to the reference category with the highest n (gender: *n* = 3, age: *n* = 9, migration background: *n* = 9, weekly physical activities: *n* = 11, subjective health status: *n* = 7) if they were under 5% [42]; otherwise they are managed as a separate category. Missings in the dependent variable (*n* = 3) were excluded in all analysis.

## Results

### Participant characteristics

Table 1 shows that the study involved 646 tenth-grade students from nine secondary schools in Witten, Germany. The gender distribution was nearly even, with 47.1% boys, 47.6% girls, and 5.3% identifying as diverse. Most students were 15 or 16 years old, making up 91.2% of the participants, while the remaining were aged 17 to 19. In terms of school type, students attended academic high school (35.4%), Intermediate secondary school (27%), comprehensive school (32%), and general secondary school (5.6%).

**Table 1:** Participants’ breakfast consumption according to sociodemographics, school form, medical history, and lifestyle characteristics (GeWIT, *n* = 646):

	Study population (total)		Had breakfast before school		Did not have breakfast before school		*P*-Value
	n	%	n	%	n	%	
**Total**	646	100%	319	49.4%*	327	50.6%*	
**Gender**							0.22
Girls	308	47.6%	147	47.7%	161	52.3%	
Boys	304	47.1%	159	52.3%	145	47.7%	
Diverse	34	5.3%	13	38.2%	21	61.8%	
**Age (in years)**							0.40
15²	383	59.3%	197	51.4%	186	48.6%	
16	206	31.9%	94	45.6%	112	54.4%	
17–19	57	8.8%	28	49.1%	29	50.9%	
**School type**							< 0.001
Academic high school	229	35.4%	138	60.3%	91	39.7%	
Intermediate secondary school	174	27%	71	40.8%	103	59.2%	
Comprehensive school	207	32%	95	45.9%	112	54.1%	
General secondary school	36	5.6%	15	41.7%	21	58.3%	
**Subjective SES**							0.29
High	331	51.2%	172	52.0%	159	48.0%	
Moderate	222	34.4%	109	49.1%	113	50.9%	
Low	49	7.6%	19	38.8%	30	61.2%	
Missing Data	44	6.8%	19	43.2%	25	56.8%	
**Migration background**							0.001
No³	357	55.3%	197	55.2%	160	44.8%	
Yes	289	44.7%	122	42.2%	167	57.8%	
**Physical activity**							0.43
≥ 3 days/week⁴	504	78.0%	253	50.2%	251	49.8%	
1–2 days/week	120	18.6%	58	48.3%	62	51.7%	
0 days/week	22	3.4%	8	36.4%	14	63.6%	
**BMI**							0.019
Underweight	34	5.2%	23	67.6%	11	32.4%	
Normal weight	337	52.2%	173	51.3%	164	48.7%	
Overweight	33	5.1%	17	51.5%	16	48.5%	
Obesity	32	5.0%	9	28.1%	23	71.9%	
Missing data	210	32.5%	97	46.2%	113	53.8%	
**Family doctor consultations last year**							0.609
0–2/ year	126	19.5%	67	53.2%	59	46.8%	
3–5/year	65	10.1%	31	47.7%	34	52.3%	
≥ 6 / year	402	62.2%	192	47.8%	210	52.2%	
Missing data	53	8.2%	29	54.7%	24	45.3%	
**Subjective health status**							< 0.001
Excellent	98	15.2%	69	70.4%	29	29.6%	
Very good	201	31.1%	108	53.7%	93	46.3%	
Good⁵	280	43.3%	118	42.1%	162	57.9%	
Less good/ bad	67	10.4%	24	35.8%	43	64.2%	

- *Row Percentages

- Subjective SES: Subjective socioeconomic status

- Percentage figures do not always add up to 100% due to rounding

- (n) does not equal the total number due to missing data; The superscript numbers refer to the included missing data: ( gender: *n* = 3, ² age: *n* = 9, ³ migration background *n* = 9, ⁴ weekly physical activities *n* = 11, ⁵ subjective health status *n* = 7)

### Breakfast intake on the day of the survey

Of all participants, 49.4% (*n* = 319) reported having breakfast before school, while 50.6% (*n* = 327) did not (Table 1). The bivariate analysis, shown in Table 1, revealed significant differences in breakfast consumption based on school type. Students from intermediate secondary school, comprehensive schools, and general secondary school had a higher tendency to skip breakfast with 59.2% (*n* = 103), 54.1% (*n* = 112), and 58.3% (*n* = 21) respectively, compared to students from academic high school who were less likely to skip breakfast, with 39.7% (*n* = 91).

Migration background also showed a significant association with breakfast skipping, with 57.8% (*n* = 167) of students with a migration background skipping breakfast, compared to 44.8% (*n* = 160) of breakfast skippers without a migration background. BMI categories were also significantly associated with breakfast skipping behaviours on the surveyed day. Specifically, among the study participants, 48.7% of normal-weight students (*n* = 164), 48.5% of overweight students (*n* = 16), and 71.9% of obese students (*n* = 23) reported skipping breakfast. In comparison, a smaller percentage of underweight students, 32.4% (*n* = 11), skipped breakfast before school.

Similarly, subjective health status was also significantly correlated with breakfast skipping. Those who rated their health as “very good” had a breakfast skipping rate of 46.3% (*n* = 93), followed by those rating their health as “good” with an amount of 57.9% (*n* = 162), and those describing their health as “less good/bad” with the highest amount of 64.2% (*n* = 43). In comparison, students labelling their health as “excellent” demonstrated a lower tendency to skip breakfast, with 29.6% (*n* = 29) reporting they did not have breakfast before school.

The results of the multiple logistic regression analysis are shown in Table 2. Several key predictors of breakfast skipping have been identified. Notably, school type, migration background, subjective health status and being underweight were significant predictors. Compared to students from academic high schools, those from intermediate secondary schools, (aOR = 1.66, 95% CI: 1.1–2.51) and comprehensive schools (aOR = 1.62, 95% CI: 1.11–2.36) had higher odds of skipping breakfast. Students with a migration background had 1.46 times the odds of skipping breakfast compared to their counterparts without a migration background (aOR = 1.46, 95% CI: 1.04–2.04). Moreover, subjective health status was significantly associated with breakfast consumption. Students who rated their health as “excellent” were less likely to skip breakfast, with an adjusted odds ratio (aOR) of 0.29, (95% CI: 0.17–0.48). Similarly, those who rated their health as “very good” had an aOR of 0.63 (95% CI: 0.43–0.91), indicating they were also less likely to skip breakfast compared to those with lower health ratings. Body Mass Index-categories (BMI) were significantly connected to breakfast skipping, showing a pronounced trend of less frequent breakfast skipping among underweight individuals (aOR = 0.44, 95% CI: 0.2–0.98). No significant associations were found in the multivariate model for gender, age, socioeconomic status, physical activity frequency, or the frequency of family doctor’s consultations with breakfast skipping.

**Table 2:** Predictors of skipping breakfast: significance, aOR, 95% CI from logistic regression, (GeWIT, *n* = 646):

	aOR (95% CI)	*P*-Value
**Gender**		0.59
Girls (Ref)		
Boys	0.97 (0.68–1.38)	0.87
Diverse	1.46 (0.67–3.21)	0.34
**Age (in years)**		0.76
15² (Ref)		
16	1.1 (0.76–1.57)	0.62
17–19	0.88 (0.48–1.6)	0.67
**School type**		0.03
Academic high school (Ref)		
Intermediate secondary school	1.66 (1.1–2.51)	0.02
Comprehensive school	1.62 (1.11–2.36)	0.01
General secondary school	1.42 (0.66–3.05)	0.37
**Subjective SES**		0.68
High (Ref)		
Moderate	0.80 (0.56–1.15)	0.23
Low	0.84 (0.43–1.64)	0.60
Missing data	0.95 (0.48–1.88)	0.88
**Migration background**		
No³ (Ref)		
Yes	1.46 (1.04–2.04)	0.03
**Physical activity**		0.67
≥ 3 days/week⁴ (Ref)		
1–2 days/week	0.92 (0.61–1.40)	0.70
0 days/week	1.45 (0.57–3.74)	0.44
**BMI**		0.12
Normal weight (Ref)		
Underweight	0.44 (0.2–0.98)	0.04
Overweight	0.87 (0.4–1.87)	0.71
Obesity	1.94 (0.84–4.5)	0.12
Missing data	1.1 (0.77–1.55)	0.63
**Family doctor consultations last year**		0.77
≥ 6 / year (Ref)		
3–5/year	0.93 (0.54–1.6)	0.80
0–2/ year	0.87 (0.57–1.31)	0.49
Missing data	0.76 (0.41–1.4)	0.36
**Subjective health status**		< 0.001
good⁵ (Ref)		
Very good	0.63 (0.43–0.91)	0.02
Excellent	0.29 (0.17–0.48)	< 0.001
Less good/ bad	1.26 (0.71–2.22)	0.43

- Adjusted Odds Ratios from binary logistic regression analysis, missing values in the independent variables were assigned to the reference category (largest n) if under 5% (*n* = 646)

- The superscript numbers refer to the included missing data: ( Gender: *n* = 3, ² Age: *n* = 9, ³ Migration background *n* = 9, ⁴ Weekly physical activities *n* = 11, ⁵ Subjective health status *n* = 7)

- Ref.= Reference category. -

- Subjective SES: Subjective socioeconomic status

## Discussion

### Interpretation of key findings

Our research revealed that 50.6% of 10th-grade adolescents did not have breakfast before school on the day of the survey, a figure that is notably high and warrants attention due to its potential implications on health and learning capacities. However, as this is a one-day cross-sectional measure, it does not necessarily reflect habitual breakfast behaviour, and further research is needed to assess long-term patterns and their impact. This concern is echoed in the study by Bucksch et al. in 2020 [6], which explored dietary habits and physical activity trends among 11-, 13-, and 15-year-olds in Germany, utilizing the 2017/18 Health Behaviour in School-aged Children (HBSC) data and comparing it to data from 2009/10 to 2013/14 cycles. Their findings document a progressive decline in daily breakfast consumption among students. Specifically, the proportion of girls having breakfast decreased successively from 63.6 to 50.6%, and further to 49.4%, while for boys, the rates declined from 67.3 to 59.0%, and subsequently to 41% over these periods. This finding aligns with existing studies that underscore the prevalence of skipped breakfasts among adolescents worldwide and it’s adverse effects on cognitive and physical development [8–10, 33]. Notably, skipping breakfast could compromise the replenishment of depleted energy stores and essential nutrient levels after overnight fasting, which is critical for daily function and long-term health [11]. These findings underscore the need for targeted health interventions to address this widespread issue.

The logistic regression analysis highlighted school type as a significant predictor of breakfast skipping, with students from Intermediate secondary schools and comprehensive schools showing increased odds of skipping breakfast compared to their academic high school counterparts. In Germany, the secondary school system is divided into academic, intermediate, comprehensive, and general secondary schools, each serving distinct student populations [43]. Academic secondary schools, which prepare students for university, are typically attended by adolescents from higher socioeconomic backgrounds and often provide a more structured academic setting with greater emphasis on health education. In contrast, intermediate and general secondary schools focus on practical or vocational education and are more commonly attended by students from lower socioeconomic backgrounds, while comprehensive schools combine elements of all tracks and serve more diverse populations [44]. Although socioeconomic status did not emerge as a significant independent predictor in our analysis, school type remains closely tied to family background, parental education, and household income. These structural and contextual differences can influence dietary behaviour through varied access to food, parental support, and morning routines. Thus, the observed association between school type and breakfast skipping likely reflects broader institutional and socio-cultural disparities that shape adolescents’ eating habits.

These insights align with previous research underscoring the role of school and educational settings in influencing health behaviours among adolescents [7–9, 20]. Schools play a crucial role in improving children’s health and are ideal for public health interventions at the population level, given that children spend approximately 40% of their weekdays in school [45]. Besides the home, school is where adolescents are mostly found, and they consume as much as half of their daily calories there [46]. This makes schools an ideal setting to encourage healthy eating habits that can last into adulthood, underlining the importance of schools and school-based nutrition programs as a key location for health-related interventions [7, 47, 48].

Additionally, the observation that adolescents from migrant backgrounds have a higher likelihood of not eating breakfast highlights the impact of socio-cultural variables on dietary behaviours on the surveyed day. This is in line with existing research that suggests difficulties related to migration, such as economic difficulties, communication barriers, social isolation and the stress of adapting to a new culture can negatively influence dietary behaviours [49–51]. Such insights are critical for designing culturally sensitive interventions that address these unique challenges.

The analysis also uncovers that students who described their general health as “excellent” and “very good” were significantly less likely to skip breakfast on the surveyed day, indicating a link between health consciousness and dietary behaviour [18]. This finding is consistent with the notion that health awareness can motivate healthier eating choices, including the adherence to regular breakfast consumption, as suggested by earlier research [9, 10]. Integrating health education that reinforces the importance of breakfast might enhance these perceptions and encourage consistent dietary patterns. Although self-rated health is a subjective indicator and does not directly measure health awareness or health knowledge, prior research suggests it is often associated with engagement in health-promoting behaviours such as physical activity, sleep hygiene, and regular meals. Therefore, while causality cannot be inferred due to the cross-sectional design, the association may reflect broader patterns of health-related behaviour among adolescents [52–54].

Furthermore, our study revealed that underweight individuals are significantly less likely to skip breakfast compared to those of normal weight, possibly due to a conscious effort to maintain or gain weight. This underscores the critical role of regular breakfast consumption in ensuring adequate nutrient intake and preventing further weight loss and potential health issues like malnutrition [25, 28, 29]. It reflects patterns noted in previous research, which emphasizes the importance of regular meals in managing weight and overall nutritional well-being [11].

The observed associations between school type, migration background, and breakfast skipping behaviour on the surveyed day reflect the complex interplay of educational, socioeconomic, and cultural factors in dietary behaviours on the surveyed day, as reported in earlier studies [8–10, 18, 21, 23, 55]. Our study adds to this body of knowledge by quantifying the odds of breakfast skipping among different school types and migrant backgrounds, providing concrete evidence for targeted interventions. The significant correlation between subjective health perception and breakfast consumption offers a valuable addition to the literature. Previous research has also suggested a link between health awareness and dietary habits [1, 4, 9, 10, 33, 55].

This study provides novel insights into breakfast consumption behaviours among German adolescents by identifying structural and psychosocial predictors, including school type and subjective health perception. While previous studies have established general associations between breakfast skipping and various sociodemographic factors, our findings offer population-specific data that are relevant for tailoring nutrition interventions within the German school system. In particular, the identification of students from non-academic school tracks and those with a migration background as high-risk groups underscores the need for targeted policies and programs. Our study not only confirms these associations but also provides quantifiable evidence of these relationships among adolescents, emphasizing the potential of health education in promoting regular breakfast consumption and supporting overall adolescent health and well-being. Nevertheless, the underlying reasons for skipping breakfast remain unclear and may include physiological, cultural, financial, or organizational factors, which warrant further investigation.

### Limitations

The data for this study was collected from schools in the city of Witten in Germany, which may limit the generalizability of the findings to other regions of the country. Furthermore, two special needs schools and two Waldorf schools were not included in the study. This exclusion may have affected the results and reduced the diversity of the sample. Although efforts were made to standardize the data collection process by maintaining a quiet environment during questionnaire completion and ensuring that students had enough privacy to respond individually, it is not possible to fully eliminate the influence of external factors. There may have been peer influence or a tendency to respond in a socially desirable way. A small number of students were removed from the dataset due to clearly inconsistent or unserious responses, which were regarded as active non-participation.

Furthermore, the length of the questionnaire may have affected data quality, as its comprehensiveness could lead to respondent fatigue. This may have influenced the accuracy or completeness of some responses, particularly toward the end of the survey.

Additionally, BMI was calculated based on self-reported height and weight, which may be subject to reporting bias and should be interpreted with caution. A considerable portion of BMI data was missing (32.5%), probably because some students chose not to report their height and/or weight, possibly due to privacy concerns or body image sensitivity. This level of missing data may have introduced selection bias, as those who refrained from reporting their measurements might systematically differ in health status or eating behaviours from those who did. Consequently, our findings regarding BMI and its association with breakfast consumption should be interpreted with caution. Future studies should consider using objective anthropometric measurements to enhance data reliability.

Another notable limitation relates to how breakfast consumption was assessed. The survey asked whether students had eaten breakfast before going to school on the day the data were collected. While this approach aimed to capture any form of morning food intake regardless of where or what was consumed, it did not distinguish between different types or amounts of food, such as a full meal versus a small snack. It also did not consider whether students had access to breakfast at school or ate later in the morning. Since the data were based on a single day, the results reflect only one specific moment rather than consistent eating habits over time. These constraints limit the ability to draw conclusions about regular breakfast behaviour and should be considered when interpreting the study’s findings. To gain a more accurate understanding of regular breakfast patterns among adolescents, future studies should include multi-day dietary assessments or adopt a longitudinal approach that captures eating behaviour over time and its potential effects on health. Moreover, while physical activity was assessed as a lifestyle characteristic, other potentially relevant behaviours such as sleep patterns, screen time, and dietary diversity were not captured. Future studies should include a broader range of lifestyle indicators to better contextualize breakfast behaviour.

## Conclusions

This study adds to the limited body of evidence on breakfast behaviours among German adolescents and underscores the importance of addressing social, structural, and health-related disparities in dietary patterns. Our findings identify specific at-risk groups, such as students from non-academic secondary schools, adolescents with a migration background, and those reporting poor self-rated health, who were more likely to have skipped breakfast on the surveyed day. While these associations offer valuable insights for school-based public health interventions, they should be interpreted within a broader nutritional and social context. Rather than promoting breakfast in isolation, public health strategies should aim to foster comprehensive and balanced dietary practices throughout the day. Schools represent a key setting for such interventions. In addition to implementing free or subsidized breakfast programs and integrating culturally sensitive nutrition education into the school curriculum, more comprehensive approaches are recommended. These could include the introduction of flexible school start times to allow students sufficient time for morning routines, the establishment of breakfast clubs to normalize and encourage morning meals through shared social experiences, and the inclusion of breakfast-related topics in health and science curricula to improve knowledge and awareness. Further, strengthening parental engagement, particularly in migrant or socioeconomically disadvantaged families through targeted outreach and educational materials could enhance support for healthy breakfast habits at home. Digital health tools and mobile applications may also be used to encourage adolescents to develop and maintain regular breakfast routines. At the policy level, expanded funding and institutional support are needed to implement these interventions sustainably and equitably across different school types and communities. By identifying groups with a higher prevalence of breakfast skipping, this study provides concrete evidence to inform tailored interventions.

The observed association between self-rated health and breakfast skipping may reflect underlying behavioural patterns, adolescents who report better general health may be more likely to engage in health-promoting behaviours, including regular meals. However, this does not imply a causal relationship or increased health awareness. As self-perception of health is a subjective indicator influenced by multiple factors, future research should explore its role alongside more objective measures of health knowledge and behaviour.

Future research should also employ longitudinal and multi-method designs to explore causal relationships and better understand the complex interplay of social, behavioural, and environmental factors influencing adolescent nutrition. Broader geographical and cultural replication of this work would further support the development of inclusive, equity-focused public health strategies.

## Electronic supplementary material

Below is the link to the electronic supplementary material.

Supplementary Material 1

## Data Availability

The data generated and analyzed during this study are not publicly accessible due to data protection reasons. However, they can be requested from the study management by contacting Prof. Dr. Eva Münster at (eva.muenster@uni-wh.de), upon reasonable request.

## Abbreviations

aOR: Adjusted Odds Ratios
Ref.: Reference category
CI: Confidence Intervals
DESTATIS: Das statistische Bundesamt (The Federal Statistical Office of Germany)
GeWIT: Gesunde Stadt Witten (Healthy City Witten)
SPSS: Statistical Package für Social Sciences
SES: Socioeconomic status
BMI: Body-Mass-Index
DDS: Diet Diversity Score
HBSC: Health Behaviour in School-age Children
KIGGS study: Studie zur Gesundheit von Kindern und Jugendlichen in Deutschland (Study on the Health of Children and Adolescents in Germany)
RKI: Robert Koch Institute (the official public health institute of Germany)

## References

[1] Scaglioni S, De Cosmi V, Ciappolino V, Parazzini F, Brambilla P, Agostoni C. Factors influencing children’s eating behaviours. Nutrients. 2018;10(6):706. 10.3390/nu10060706. PMID: 29857549; PMCID: PMC6024598. 29857549 10.3390/nu10060706 PMC6024598

[2] Sorrenti V, Burò I, Consoli V, Vanella L. Recent advances in health benefits of bioactive compounds from food wastes and By-Products: biochemical aspects. Int J Mol Sci. 2023;24(3):2019. 10.3390/ijms24032019. PMID: 36768340; PMCID: PMC9916361. 36768340 10.3390/ijms24032019 PMC9916361

[3] Bowen KJ, Sullivan VK, Kris-Etherton PM, Petersen KS. Nutrition and Cardiovascular Disease-an Update. Curr Atheroscler Rep. 2018;20(2):8. 10.1007/s11883-018-0704-3. PMID: 29383458. 10.1007/s11883-018-0704-3 29383458

[4] Brettschneider AK, Lage Barbosa C, Haftenberger M, Lehmann F, Mensink GB. Adherence to food-based dietary guidelines among adolescents in Germany according to socio-economic status and region: results from eating study as a KiGGS module (EsKiMo) II. Public Health Nutr. 2021;24(6):1216–28. Epub 2021 Jan 11. PMID: 33427143; PMCID: PMC8025090. 33427143 10.1017/S136898002100001X PMC8025090

[5] Sichert-Hellert W, Beghin L, De Henauw S, Grammatikaki E, Hallström L, Manios Y, Mesana MI, Molnár D, Dietrich S, Piccinelli R, Plada M, Sjöström M, Moreno LA, Kersting M, HELENA Study Group. Nutritional knowledge in European adolescents: results from the HELENA (Healthy lifestyle in Europe by nutrition in Adolescence) study. Public Health Nutr. 2011;14(12):2083–91. 10.1017/S1368980011001352. Epub 2011 Aug 2. PMID: 21810282. 21810282 10.1017/S1368980011001352

[6] Bucksch J, Häußler A, Schneider K, Finne E, Schmidt K, Dadacynski K, Sudeck G. Physical activity and dietary habits of older children and adolescents in Germany - Cross-sectional results of the 2017/18 HBSC study and trends. J Health Monit. 2020;5(3):21–36. 10.25646/6900. PMID: 35146271; PMCID: PMC8734148. 35146271 10.25646/6900 PMC8734148

[7] Devine LD, Hill AJ, Gallagher AM. Improving adolescents’ dietary behaviours in the school-setting: challenges and opportunities. Proc Nutr Soc. 2023;82(2):172–85. Epub 2023 Feb 14. PMID: 36916515. 36916515 10.1017/S0029665123002197

[8] Rampersaud GC, Pereira MA, Girard BL, Adams J, Metzl JD. Breakfast habits, nutritional status, body weight, and academic performance in children and adolescents. J Am Diet Assoc. 2005;105(5):743– 60; quiz 761-2. 10.1016/j.jada.2005.02.007. PMID: 15883552. 10.1016/j.jada.2005.02.007 15883552

[9] Van Lippevelde W, Te Velde SJ, Verloigne M, Van Stralen MM, De Bourdeaudhuij I, Manios Y, Bere E, Vik FN, Jan N, Fernández Alvira JM, Chinapaw MJ, Bringolf-Isler B, Kovacs E, Brug J, Maes L. Associations between family-related factors, breakfast consumption and BMI among 10- to 12-year-old European children: the cross-sectional ENERGY-study. PLoS ONE. 2013;8(11):e79550. 10.1371/journal.pone.0079550. PMID: 24282508; PMCID: PMC3840060. 24282508 10.1371/journal.pone.0079550 PMC3840060

[10] Baldinger N, Krebs A, Müller R, Aeberli I. Swiss children consuming breakfast regularly have better motor functional skills and are less overweight than breakfast skippers. J Am Coll Nutr. 2012;31(2):87–93. doi: 10.1080/07315724.2012.10720013. PMID: 22855913. 10.1080/07315724.2012.10720013 22855913

[11] Adonu RE, Amoah M, Saah FI. Breakfast intake and associated factors and barriers among tertiary institution students in the Western region, Ghana. BMC Nutr. 2023;9(1):7. 10.1186/s40795-023-00672-6. PMID: 36627687; PMCID: PMC9830603. 36627687 10.1186/s40795-023-00672-6 PMC9830603

[12] de la Hunty A, Gibson S, Ashwell M. Does regular breakfast cereal consumption help children and adolescents stay slimmer? A systematic review and meta-analysis. Obes Facts. 2013;6(1):70–85. doi: 10.1159/000348878. Epub 2013 Mar 2. PMID: 23466487; PMCID: PMC5644749. 10.1159/000348878 PMC5644749 23466487

[13] O’Neil CE, Byrd-Bredbenner C, Hayes D, Jana L, Klinger SE, Stephenson-Martin S. The role of breakfast in health: definition and criteria for a quality breakfast. J Acad Nutr Diet. 2014;114(12 Suppl):S8–26. Epub 2014 Nov 24. PMID: 25458994. 25458994 10.1016/j.jand.2014.08.022

[14] Simeon DT. School feeding in Jamaica: a review of its evaluation. Am J Clin Nutr. 1998;67(4):790S-794S. 10.1093/ajcn/67.4.790S. PMID: 9537630. 10.1093/ajcn/67.4.790S 9537630

[15] Pollitt E, Mathews R. Breakfast and cognition: an integrative summary. Am J Clin Nutr. 1998;67(4):804S-813S. 10.1093/ajcn/67.4.804S. PMID: 9537633. 10.1093/ajcn/67.4.804S 9537633

[16] Powell CA, Walker SP, Chang SM, Grantham-McGregor SM. Nutrition and education: a randomized trial of the effects of breakfast in rural primary school children. Am J Clin Nutr. 1998;68(4):873-9. 10.1093/ajcn/68.4.873. PMID: 9771865. 10.1093/ajcn/68.4.873 9771865

[17] Jacoby E, Cueto S, Pollitt E. Benefits of a school breakfast programme among Andean children in Huaraz, Peru. FoodNutr Bull. 1996;17(1):1–11. 10.1177/156482659601700111.

[18] Kesztyüs D, Traub M, Lauer R, et al. Skipping breakfast is detrimental for primary school children: cross-sectional analysis of determinants for targeted prevention. BMC Public Health. 2017;17:258. 10.1186/s12889-017-4169-z. 28292281 10.1186/s12889-017-4169-z PMC5351158

[19] Ma X, Chen Q, Pu Y, Guo M, Jiang Z, Huang W, Long Y, Xu Y. Skipping breakfast is associated with overweight and obesity: A systematic review and meta-analysis. Obes Res Clin Pract. 2020 Jan-Feb;14(1):1–8. Epub 2020 Jan 7. PMID: 31918985. 10.1016/j.orcp.2019.12.002 31918985

[20] Badrasawi M, Anabtawi O, Al-Zain Y. Breakfast characteristics, perception, and reasons of skipping among 8th and 9th-grade students at governmental schools, Jenin governance, West bank. BMC Nutr. 2021;7(1):42. 10.1186/s40795-021-00451-1. PMID: 34353371; PMCID: PMC8342035. 34353371 10.1186/s40795-021-00451-1 PMC8342035

[21] Tin SP, Ho SY, Mak KH, Wan KL, Lam TH. Lifestyle and socioeconomic correlates of breakfast skipping in Hong Kong primary 4 schoolchildren. Prev Med. 2011 Mar-Apr;52(3–4):250-3. doi: 10.1016/j.ypmed.2010.12.012. Epub 2011 Jan 4. PMID: 21215276. 10.1016/j.ypmed.2010.12.012 21215276

[22] Tabrizi JS, Doshmangir L, Khoshmaram N, Shakibazadeh E, Abdolahi HM, Khabiri R. Key factors affecting health promoting behaviors among adolescents: a scoping review. BMC Health Serv Res. 2024;24(1):58. 10.1186/s12913-023-10510-x. PMID: 38212786; PMCID: PMC10782684. 38212786 10.1186/s12913-023-10510-x PMC10782684

[23] Szajewska H, Ruszczynski M. Systematic review demonstrating that breakfast consumption influences body weight outcomes in children and adolescents in Europe. Crit Rev Food Sci Nutr. 2010;50(2):113-9. 10.1080/10408390903467514. PMID: 20112153. 10.1080/10408390903467514 20112153

[24] Freitas Júnior IF, Christofaro DG, Codogno JS, Monteiro PA, Silveira LS, Fernandes RA. The association between skipping breakfast and biochemical variables in sedentary obese children and adolescents. J Pediatr. 2012;161(5):871-4. 10.1016/j.jpeds.2012.04.055. Epub 2012 Jun 7. PMID: 22682613. 10.1016/j.jpeds.2012.04.055 22682613

[25] Chitra U, Reddy CR. The role of breakfast in nutrient intake of urban schoolchildren. Public Health Nutr. 2007;10(1):55– 8. doi: 10.1017/S1368980007219640. PMID: 17212843. 10.1017/S1368980007219640 17212843

[26] Fisberg M, Kovalskys I, Previdelli AN, Pereira JL, Zimberg IZ, Fisberg R, Ferrari G, Guajardo V, The Elans Study Group. Breakfast consumption habit and its nutritional contribution in Latin America: results from the ELANS study. Nutrients. 2020;12(8):2397. 10.3390/nu12082397. PMID: 32785188; PMCID: PMC7468943. 32785188 10.3390/nu12082397 PMC7468943

[27] TY H, Krawinkel M. Dietary diversity score: A measure of nutritional adequacy or an Indicator of healthy diet?? J Nutr Health Sci. 2016;3. 10.15744/2393-9060.3.303.

[28] Iizuka, Katsumi & Sato, Hiroko & Kobae, Kazuko & Yanagi, Kotone & Yamada, Yoshiko& Ushiroda, Chihiro & Hirano, Konomi & Ichimaru, Satomi & Seino, Yusuke & Ito, Akemi& Suzuki, Atsushi & Saitoh, Eiichi & Naruse, Hiroyuki. (2023). Young Japanese Underweight Women with “Cinderella Weight” Are Prone to Malnutrition, including Vitamin Deficiencies.Nutrients. 15. 2216. 10.3390/nu15092216. 10.3390/nu15092216 PMC10181057 37409654

[29] Smith KJ, Breslin MC, McNaughton SA, Gall SL, Blizzard L, Venn AJ. Skipping breakfast among Australian children and adolescents; findings from the 2011-12 National nutrition and physical activity survey. Aust N Z J Public Health. 2017;41(6):572–8. Epub 2017 Sep 12. PMID: 28898562. 28898562 10.1111/1753-6405.12715

[30] Frank M, Brettschneider A-K, Lage Barbosa C, Haftenberger M, Lehmann F, Perlitz H, Heide K, Patelakis E, Richter A, Mensink GBM. Prevalence and Temporal trends of shared family meals in Germany. Results from EsKiMo II. Ernahrungs Umschau. 2019;66(4):60–7. 10.4455/eu.2019.013.

[31] Hammons AJ, Fiese BH. Is frequency of shared family meals related to the nutritional health of children and adolescents? Pediatrics. 2011;127(6):e1565–74. 10.1542/peds.2010-1440. Epub 2011 May 2. PMID: 21536618; PMCID: PMC3387875. 21536618 10.1542/peds.2010-1440 PMC3387875

[32] Alexy U, Wicher M, Kersting M. Breakfast trends in children and adolescents: frequency and quality. Public Health Nutr. 2010;13(11):1795– 802. doi: 10.1017/S1368980010000091. Epub 2010 Mar 18. PMID: 20236559. 10.1017/S1368980010000091 20236559

[33] Timlin MT, Pereira MA, Story M, Neumark-Sztainer D. Breakfast eating and weight change in a 5-year prospective analysis of adolescents: Project EAT (Eating Among Teens). Pediatrics. 2008;121(3):e638-45. 10.1542/peds.2007-1035. PMID: 18310183. 10.1542/peds.2007-1035 18310183

[34] Vereecken C, Dupuy M, Rasmussen M, Kelly C, Nansel TR, Al Sabbah H, Baldassari D, Jordan MD, Maes L, Niclasen BV, Ahluwalia N, HBSC Eating & Dieting Focus Group. Breakfast consumption and its socio-demographic and lifestyle correlates in schoolchildren in 41 countries participating in the HBSC study. Int J Public Health. 2009;54(Suppl 2):180–90. 10.1007/s00038-009-5409-5. PMID: 19639257; PMCID: PMC3408388. 19639257 10.1007/s00038-009-5409-5 PMC3408388

[35] Bevölkerung der Gemeinden Nordrhein-Westfalens am 31. Dezember 2023– Fortschreibung des Bevölkerungsstandes auf Basis des Zensus vom 9. Mai 2011. (Population of the municipalities of North Rhine-Westphalia as of December 31, 2023– Update of the population figures based on the census of May 9, 2011).

[36] Healthy Youth Witten (GeJuWIT). A survey of students on their well-being and health behavior in Witten, conducted by the Institute of general practice and outpatient healthcare. Witten/Herdecke University; 2021.

[37] Hölling H, Kamtsiuris P, Lange M, Thierfelder W, Thamm M, Schlack R. Der Kinder- und Jugendgesundheitssurvey (KiGGS): Studienmanagement und Durchführung der Feldarbeit [The German Health Interview and Examination Survey for Children and Adolescents (KiGGS): study management and conduct of fieldwork]. Bundesgesundheitsblatt Gesundheitsforschung Gesundheitsschutz. 2007 May-Jun;50(5–6):557– 66. German. 10.1007/s00103-007-0216-8. PMID: 17514439.(The German Health Interview and Examination Survey for Children and Adolescents (KiGGS): Study Management and Conduct of Fieldwork). 10.1007/s00103-007-0216-8 17514439

[38] Winter K, Moor I, Markert J, Bilz L, Bucksch J, Dadaczynski K, Fischer SM, Helmchen RM, Kaman A, Möckel J, Rathmann K, Ravens-Sieberer U, Reiß F, Schierl T, Schütz R, Sendatzki S, Stürmer E, Sudeck G, Richter M, HBSC Study Group Germany. Concept and methodology of the health behaviour in School-aged children (HBSC) study - Insights into the current 2022 survey and trends in Germany. J Health Monit. 2024;9(1):99–117. 10.25646/11878. PMID: 38559683; PMCID: PMC10977469. 38559683 10.25646/11878 PMC10977469

[39] Statistisches Bundesamt (Destatis). Bevölkerung mit Migrationshintergrund– Ergebnisse des Mikrozensus 2020; 2022.(Federal Statistical Office (Destatis). Population with a migration background– Results of the 2020 Microcensus. 2022).

[40] Kromeyer-Hauschild K, Moss A, Wabitsch. (2015). Referenzwerte für den Body-Mass-Index für Kinder, Jugendliche und Erwachsene in Deutschland: Anpassung der AGA-BMI-Referenz im Altersbereich von 15 bis 18 Jahren. Adipositas - Ursachen, Folgeerkrankungen, Therapie. 09. 123–127. 10.1055/s-0037-1618928.(Reference values for the Body Mass Index for children, adolescents, and adults in Germany: Adjustment of the AGA BMI reference in the age range of 15 to 18 years. Obesity– Causes, Comorbidities, Therapy).

[41] Hoebel J, Müters S, Kuntz B, Lange C, Lampert T. Messung des subjektiven sozialen status in der gesundheitsforschung Mit einer Deutschen version der MacArthur scale [Measuring subjective social status in health research with a German version of the MacArthur scale]. Bundesgesundheitsblatt Gesundheitsforschung Gesundheitsschutz. 2015;58(7):749–57. German. doi: 10.1007/s00103-015-2166-x. PMID: 25986532.(Measuring subjective social status in health research using a German version of the MacArthur Scale). 25986532 10.1007/s00103-015-2166-x

[42] Ziarko W, editor. Rough Sets and Current Trends in Computing: Second International Conference, RSCTC 2000 Banff, Canada, October 1619, 2000 Revised Papers. Berlin, Heidelberg: Springer-Verlag Berlin Heidelberg; 2001. (SpringerLink Bücher; vol 2005).

[43] Ministerium für Schule und Bildung des Landes Nordrhein-Westfalen. https://www.schulministerium.nrw/.(Ministry of School and Education of the State of North Rhine-Westphalia).

[44] Maaz K, Trautwein U, Lüdtke O, Baumert Jürgen. (2008). Educational Transitions and Differential Learning Environments: How Explicit Between-School Tracking Contributes to Social Inequality in Educational Outcomes. Child Development Perspectives. 2. 99–106. 10.1111/j.1750-8606.2008.00048.x

[45] Woodside JV, Adamson A, Spence S, Baker T, McKinley MC. GENIUS (Generating excellent nutrition in UK Schools) network. Opportunities for intervention and innovation in school food within UK schools. Public Health Nutr. 2021;24(8):2313–7. Epub 2020 Nov 17. PMID: 33198839; PMCID: PMC10195578. 33198839 10.1017/S1368980020004668 PMC10195578

[46] Micha R, Karageorgou D, Bakogianni I, Trichia E, Whitsel LP, Story M, Peñalvo JL, Mozaffarian D. Effectiveness of school food environment policies on children’s dietary behaviors: A systematic review and meta-analysis. PLoS ONE. 2018;13(3):e0194555. 10.1371/journal.pone.0194555. PMID: 29596440; PMCID: PMC5875768. 29596440 10.1371/journal.pone.0194555 PMC5875768

[47] Chortatos A, Terragni L, Henjum S, Gjertsen M, Torheim LE, Gebremariam MK. Consumption habits of school canteen and non-canteen users among Norwegian young adolescents: a mixed method analysis. BMC Pediatr. 2018;18(1):328. 10.1186/s12887-018-1299-0. PMID: 30326859; PMCID: PMC6192152. 30326859 10.1186/s12887-018-1299-0 PMC6192152

[48] Egg S, Wakolbinger M, Reisser A, Schätzer M, Wild B, Rust P. Relationship between nutrition knowledge, education and other determinants of food intake and lifestyle habits among adolescents from urban and rural secondary schools in Tyrol, Western Austria. Public Health Nutr. 2020;23(17):3136–47. Epub 2020 Jul 17. PMID: 32677602; PMCID: PMC7708993. 32677602 10.1017/S1368980020000488 PMC7708993

[49] Bhugra D. Migration, distress and cultural identity. Br Med Bull. 2004;69:129– 41. 10.1093/bmb/ldh007. PMID: 15226202. 10.1093/bmb/ldh007 15226202

[50] Sanou D, O’Reilly E, Ngnie-Teta I, Batal M, Mondain N, Andrew C, Newbold BK, Bourgeault IL. Acculturation and nutritional health of immigrants in Canada: a scoping review. J Immigr Minor Health. 2014;16(1):24–34. 10.1007/s10903-013-9823-7. PMID: 23595263; PMCID: PMC3895180. 23595263 10.1007/s10903-013-9823-7 PMC3895180

[51] Lee SD, Kellow NJ, Huggins CE, Choi TST. How and why diets change Post-Migration: A qualitative exploration of dietary acculturation among recent Chinese immigrants in Australia. Nutrients. 2022;14(17):3573. 10.3390/nu14173573. PMID: 36079830; PMCID: PMC9460769. 36079830 10.3390/nu14173573 PMC9460769

[52] Gonzalez-Alvarez A, Kimmel KA, Rosenkranz SK, Mailey E, Rosenkranz RR. Are lifestyle behaviors associated with excellent self-rated health among American adolescents? A cross-sectional study. J Healthy Eat Act Living. 2023;3(3):112–23. PMID: 38344455; PMCID: PMC10854956. 38344455 PMC10854956

[53] Page R, Simonek J, Ihász F, Hantiu, IacobUvacsek, MartinaDr. Kalabiska, Irina, Klarova R. (2009). Self-rated health, psychosocial functioning, and other dimensions of adolescent health in Central and Eastern European adolescents. European Journal of Psychiatry - EUR J PSYCHIAT. 23. 10.4321/S0213-61632009000200004

[54] Zhang T, Lu G, Wu XY. Associations between physical activity, sedentary behaviour and self-rated health among the general population of children and adolescents: a systematic review and meta-analysis. BMC Public Health. 2020;20(1):1343. 10.1186/s12889-020-09447-1. PMID: 32883275; PMCID: PMC7650260. 32883275 10.1186/s12889-020-09447-1 PMC7650260

[55] Sincovich A, Moller H, Smithers L, Brushe M, Lassi ZS, Brinkman SA, Gregory T. Prevalence of breakfast skipping among children and adolescents: a cross-sectional population level study. BMC Pediatr. 2022;22(1):220. 10.1186/s12887-022-03284-4. PMID: 35459164; PMCID: PMC9034546. 35459164 10.1186/s12887-022-03284-4 PMC9034546

