# Correction: Rise and dine: unraveling breakfast habits among tenth graders - a cross-sectional study among 646 students in the City of Witten, Germany (GeWIT study)

**DOI:** 10.1186/s12889-026-27163-0

**Published:** 2026-04-16

**Authors:** Mohamed Salah Anwar Mohamed, Nessia Rachma Dianti, Anne-Lisa Heye, Klaus Völkel, Judith Tillmann, Oxana Klassen, Paul Wiesheu, Klaus Weckbecker, Eva Münster

**Affiliations:** 1 Institute for General Medicine and Primary Care, Chair of General Medicine I and Interprofessional Care, University of Witten/Herdecke, University of Witten/Herdecke, Alfred-Herrhausen-Street 50, 58455 Witten, Germany; 2 Institute for General Medicine and Primary Care, Chair of General Medicine II and Patient-Centredness in Primary Care, University of Witten/ Herdecke, Witten, Germany; 3 Office for Labor, Health Economy, Technology Transfer, and University Development, Witten, Germany

**Correction: BMC Public Health 25**,** 1789 (2025)**

**https://doi.org/10.1186/s12889-025-23002-w**

Following publication of the original article [1], an error was identified in the author list and within the ‘Study population and recruitment, execution’ and ‘Ethics approval and consent to participate’ sections. The second author was removed, as the entry was a duplicate of the first author’s name, Mohamed Salah Anwar Mohamed.

## Study population and recruitment, execution

The paragraph currently reads:

Following approval by the Ethics Committee of the University of Witten/Herdecke (Reference No. 97/2019) and authorization by the data protection officer (Reference Nos. DT-537 and DT-630), formal permission to conduct the study was obtained from the responsible school authority of the Ennepe-Ruhr District. Participating schools were contacted in writing by staff of the conducting institute and informed about the study objectives, content, and procedures. Upon institutional approval, study information and parental consent forms were distributed to designated school contacts two weeks prior to data collection. These materials were forwarded to the students and their legal guardians.

Participation was strictly limited to students who returned a signed informed consent form from a parent or legal guardian. On the day of data collection, all students whose parental consent had been obtained were briefed again on the study’s purpose, the voluntary nature of participation, data protection measures, and their right to withdraw at any time without consequences. Verbal assent was obtained from each student prior to the administration of the questionnaire. Students who had not returned the signed consent form were not permitted to participate and were provided with alternative educational activities. The Ethics Committee confirmed that informed consent was duly obtained from both students and their legal guardians.

The paragraph should read:

Following approval by the Ethics Committee, the project was carried out in nine municipal secondary schools in Witten, Germany. The survey instrument, including the study protocol, was formally approved by the Ethics Committee in an amendment dated July 29, 2021. Before data collection started, authorization was sought by contacting the school authority of the Ennepe-Ruhr District. Subsequently, the school administrators were contacted in writing by employees of the institute conducting the study and informed about the content and purpose of the study. After permission was granted, the preparatory steps for implementation were undertaken. Two weeks before the study implementation, two separate information letters for students and parents/guardians were sent to school administrators or designated contact persons from the teaching staff, which were then forwarded to students and parents/guardians. Participating teachers were informed by the school administrators. On regular school days, data were collected in 28 classes by means of self-administered questionnaires, which students completed during lesson time in agreement with school administrators. This is intended to reach a broad coverage of the students. Prior to completion, participants were informed about the aims of the project, inclusion and exclusion criteria, voluntary participation, data protection, anonymity, and the option to withdraw at any time. Filling in and submitting the questionnaire, which required around 25 min, was considered to represent consent. Ethical approval and clearance regarding data protection were obtained from the Ethics Committee of Witten/Herdecke University (ethics approval no. 97/2019; data protection votes no. DT-537 and DT-630). The study received financial support from the statutory health insurance provider Techniker Krankenkasse.

**Ethics approval and consent to participate**

The paragraph currently reads:

The GeWIT Study received ethical approval from the Ethics Committee of Witten/Herdecke University, Germany (Approval No. 97/2019) and was conducted in accordance with the Declaration of Helsinki. Participation was voluntary, and informed consent was obtained through the completion and submission of an anonymous questionnaire by the students, with prior written consent from their legal guardians

The paragraph should read:

The GeWIT Study received ethical approval from the Ethics Committee of Witten/Herdecke University, Germany (Approval No. 97/2019) and was conducted in accordance with the Declaration of Helsinki. Participation was voluntary. Students and their parents or legal guardians received written information about the study prior to data collection. Students were informed about the study aims, voluntary participation, data protection, anonymity, and their right to withdraw at any time. Completion and submission of the anonymous questionnaire was considered to represent implied consent to participate.

The original article [1] has been corrected.
