# Supplementary material for: Rise and dine: unraveling breakfast habits among tenth graders - a cross-sectional study among 646 students in the City of Witten, Germany (GeWIT study)
Source: BMC Public Health. 2025 May 15;25:1789. doi: 10.1186/s12889-025-23002-w (PMC12079850; doi:10.1186/s12889-025-23002-w)
Supplement: Supplementary file 1 — Supplementary Material 1 [file 12889_2025_23002_MOESM1_ESM.pdf]

Healthy Youth Witten
(GeJuWIT)
Survey of students
about their well-being and health behavior in Witten.
© Institute for General Medicine and Outpatient Health Care, University of Witten/Herdecke, 2021

Note:

Please fill out this questionnaire and take part in our survey. Your information is voluntary, and you will not face any disadvantages if you choose not to participate. You can stop the survey at any time. Your data will be treated anonymously, and no conclusions can be drawn about your identity.

© Institute for General Medicine and Outpatient Health Care, University of Witten/Herdecke, 2021

I. Information about yourself

1. What is your gender?

- Female
- Male
- Diverse

2. How old are you?

- ___ years

3. In which district of Witten do you live?

- Annen
- Rüdinghausen
- Bommern
- Stockum/Düren
- Herbede
- Witten-Mitte
- Heven
- I do not live in Witten

II. Health and Exercise

4. How would you describe your overall health?
   - Excellent
   - Very good
   - Good
   - Fair
   - Poor

5. Thinking about the past week…
   - How often did you feel fit and well?
     - Not at all / A little / Moderately / Quite a bit / Very much
   - How often did you engage in physical activity (e.g., running, climbing, cycling)?
     - Not at all / A little / Moderately / Quite a bit / Very much
   - How well could you run?
     - Not at all / A little / Moderately / Quite a bit / Very much

6. Thinking about the past week…
   - How often were you full of energy?
     - Never / Rarely / Sometimes / Often / Always

III. Feelings and Moods

7. Thinking about the past week…
   - How much did you enjoy your life?
     - Not at all / A little / Moderately / Quite a bit / Very much

8. Thinking about the past week…
   - How often were you in a good mood?
     - Never / Rarely / Sometimes / Often / Always
   - How often did you have fun?

- Never / Rarely / Sometimes / Often / Always
- How often did you feel sad?
  - Never / Rarely / Sometimes / Often / Always
- How often did you feel so bad that you didn’t want to do anything?
  - Never / Rarely / Sometimes / Often / Always
- How often did you feel lonely?
  - Never / Rarely / Sometimes / Often / Always
- How often were you satisfied with yourself?
  - Never / Rarely / Sometimes / Often / Always

IV. Family and Leisure

9. Thinking about the past week…
- Did you have enough time for yourself?
  - Never / Rarely / Sometimes / Often / Always
- Could you do the things you wanted to do in your free time?
  - Never / Rarely / Sometimes / Often / Always
- Did your mother/father have enough time for you?
  - Never / Rarely / Sometimes / Often / Always
- Did your mother/father treat you fairly?
  - Never / Rarely / Sometimes / Often / Always
- Could you talk to your mother/father when you wanted to?
  - Never / Rarely / Sometimes / Often / Always
- Did you have enough money to do the same things as your friends?
  - Never / Rarely / Sometimes / Often / Always
- Did you have enough money for the things you needed?
  - Never / Rarely / Sometimes / Often / Always

V. Friends

10. Thinking about the past week…
   - Did you spend time with your friends?
     - Never / Rarely / Sometimes / Often / Always
   - Did you have fun with your friends?
     - Never / Rarely / Sometimes / Often / Always
   - Did you and your friends help each other?
     - Never / Rarely / Sometimes / Often / Always
   - Could you rely on your friends?
     - Never / Rarely / Sometimes / Often / Always

VI. School and Learning

11. Thinking about the past week…
   - Were you happy at school?
     - Not at all / A little / Moderately / Quite a bit / Very much
   - Did you get along well at school?
     - Not at all / A little / Moderately / Quite a bit / Very much

12. Thinking about the past week…
   - Were you able to pay attention?
     - Never / Rarely / Sometimes / Often / Always
   - Did you get along well with your teachers?
     - Never / Rarely / Sometimes / Often / Always

VII. Bullying

13. Have you been bullied in the last 12 months?
   - Yes / No

14. If you have been bullied, how were you bullied? (multiple answers possible)

- I have not been bullied
- Online (e.g., Instagram, TikTok, WhatsApp)
- Verbally
- Physically (e.g., pushing, hitting)
- Face to face
- Other: ________________

VIII. Nutrition

15. Did you have breakfast before school today?
   - Yes / No

16. Did you bring something to eat to school today?
   - Yes / No

17. Where do you usually eat your lunch on a school day?
   - At home
   - At school
   - At friends'/relatives' houses
   - On the go
   - Nowhere
   - Other: ________________

18. Do you like the lunch you eat on a school day?
   - Never / Rarely / Sometimes / Often / Always

19. How often have you drunk sugary energy drinks in the last four weeks?
   - Never / Once a month or less / 2-4 times a month / 2-3 times a week / 4 times a week or more

20. Where and how often do you usually meet your friends at the following places?

- Skater parks: Never / Rarely / Sometimes / Often / Always
- Parks, public green spaces: Never / Rarely / Sometimes / Often / Always
- Schoolyards: Never / Rarely / Sometimes / Often / Always
- Cafes, restaurants: Never / Rarely / Sometimes / Often / Always
- Shisha bars: Never / Rarely / Sometimes / Often / Always
- Dance schools/nightclubs/clubs: Never / Rarely / Sometimes / Often / Always
- Garden: Never / Rarely / Sometimes / Often / Always
- At home/parents' house: Never / Rarely / Sometimes / Often / Always
- At friends' houses: Never / Rarely / Sometimes / Often / Always
- Online (chatting, video calls, virtually in games): Never / Rarely / Sometimes / Often / Always
- Other (specify): _______________

21. Where do you not like to go because you don't feel safe there?

(Multiple answers possible)

- Skater parks
- Nightclubs/clubs/dance schools
- City center
- Nature/forest
- Parks, public green spaces
- Garden
- Schoolyards
- At home/parents' house
- Cafes, restaurants
- Friends' houses
- Train stations/bus stops
- Online
- Shisha bars
- Other: _______________

IX. Development

22. How tall are you without shoes?

- ___ cm

23. How much do you weigh without clothes?

- ___ kg
- I don’t know my weight
- My weight is private

24. How much pressure do you feel regarding your weight from…

- Yourself
- Parents
- Friends
- Classmates
- Instagram
- Snapchat
- TikTok
- Other: ________________

X. Health Behavior

25. Do you currently smoke?

- No / Daily / Several times a week / Once a week / Less frequently

26. At what age did you start smoking regularly?

- ___ years
- I have never smoked regularly

27. Have you ever consumed alcohol?

- Yes / No

28. How often do you drink alcoholic beverages (e.g., a glass of wine, beer, mixed drinks, spirits)?

- Never / Once a month or less / 2-4 times a month / 2-3 times a week / 4 times a week or more

29. How do you assess the health risk for people your age who engage in the following behaviors?

- Smoking cigarettes:
  - Very low / Low / Medium / High / Very high
- Smoking shisha:
  - Very low / Low / Medium / High / Very high
- Drinking alcohol:
  - Very low / Low / Medium / High / Very high
- Using cannabis:
  - Very low / Low / Medium / High / Very high
- Having unprotected sex:
  - Very low / Low / Medium / High / Very high
- Using digital media:
  - Very low / Low / Medium / High / Very high

XI. Health Knowledge

30. At what age did you learn to swim (without floatation devices)?

- ___ years
- I don't know, but I can swim without assistance
- I can't swim without assistance

31. Which of the following health apps/mobile health functions do you use?

- I don't use any health-related apps
- Calorie counter / Step tracker (e.g., running apps, fitness tracker)
- Calorie counter
- Wii/Playstation/Computer exercise program
- Online exercise programs (e.g., YouTube)

- Corona-Warn-App
- Health features of my smartwatch/Apple Watch
- Corona vaccination pass
- Other: _______________

XII. Use of Health Services

32. Please let us know which types of medical specialists you have visited in the last 12 months, and how often. (Include home visits, but not hospital or rehabilitation visits. Multiple answers possible.)

- Pediatrician
  - Yes, visited: ___ times
- General practitioner
  - Yes, visited: ___ times
- Internist (e.g., cardiologist, pulmonologist, diabetologist)
  - Yes, visited: ___ times
- Gynecologist
  - Yes, visited: ___ times
- Ophthalmologist
  - Yes, visited: ___ times
- Orthopedist
  - Yes, visited: ___ times
- Ear, Nose, and Throat doctor
  - Yes, visited: ___ times
- Neurologist
  - Yes, visited: ___ times
- Psychiatrist/Child and Adolescent Psychiatrist/Psychotherapist
  - Yes, visited: ___ times
- Psychologist or psychotherapist
  - Yes, visited: ___ times
- Surgeon
  - Yes, visited: ___ times

- Dermatologist

  - Yes, visited: ___ times

- Radiologist

  - Yes, visited: ___ times

- Urologist

  - Yes, visited: ___ times

- Dentist/Orthodontist

  - Yes, visited: ___ times

- Other doctor: ______________________

  - Yes, visited: ___ times

  - I have not visited any doctor in the last 12 months

33. Do you have a family doctor or pediatrician whom you trust?

   - Yes, I have a family doctor whom I trust

   - Yes, I have a pediatrician whom I trust

   - No, I do not have a trusted family doctor or pediatrician

34. At what age did you switch from a pediatrician to a family doctor?

   - At the age of: ___ years

   - I don't know

   - I plan to switch to a family doctor at the age of: ___ years

35. What gender should your family doctor or pediatrician be for you to feel comfortable?

   - My doctor should be the same gender as me

   - My doctor should be a different gender than me

   - I do not care about my doctor's gender

36. Was it easy for you to switch from a pediatrician to a family doctor, or will it be easy when you switch?

   - Yes / No / I don't know

37. If it wasn't or won't be easy for you to switch from a pediatrician to a family doctor, what were/are the reasons?

XIII. Situation During the Pandemic

38. If you were to feel stabbing, pressing, or wave-like pain in your lower right abdomen today, what would you do? (Multiple answers possible)

- I would go to the pediatrician
- I would go to the family doctor
- I would go to the hospital
- I would go to a naturopath
- I would ask my parents
- I would stay at home until I feel better

39. How long do you wear a mask during your school day? (Multiple answers possible)

- I wear a mask all day at school
- I wear a mask only in class
- I do not wear a mask on the schoolyard
- I wear a mask in the gym
- I never wear a mask at school
- I wear a mask during outdoor sports

40. What kind of mask do you wear during your school day?

- FFP1 mask
- FFP2 mask
- Surgical/medical mask
- Homemade mask
- I never wear a mask

41. Indicate what applies to you:

- I have/had COVID-19
- I have/had to quarantine because of COVID-19

- Distance learning has benefited me
- Distance learning has harmed me
- Wearing a mask is difficult for me
- Wearing a mask is easy for me
- I gained weight during the lockdown
- I lost weight during the lockdown
- I am afraid of COVID-19
- I have experienced more violence during the COVID-19 period
- I feel adequately informed about vaccination
- I feel adequately informed about the risks of infection, transmission, and the potential consequences of COVID-19

42. Indicate what applies to you: Through the COVID-19 vaccination…

- I protect myself
- I protect others
- I have more freedom
- I have less fear
- I have more fear
- I harm myself

43. What do you think about getting vaccinated against COVID-19?

- I have been vaccinated once
- I have been vaccinated twice
- I want to get vaccinated, but my parents do not want me to
- I want to get vaccinated, and my parents do not oppose it
- I am not interested in getting vaccinated because ____________________

44. Which preventive medical checkups have you had?

- J1 checkup (usually between the ages of 12 and 14, including blood and urine tests):
  - Yes / No
- J2 checkup (usually between the ages of 16 and 17, focusing on puberty, sexuality, and posture):

- Yes / No

- Other: _______________

  - Yes / No

- I don’t know

- I haven’t had either the J1 or J2 checkup

XIV. Exercise

45. On how many days of a typical week are you physically active for at least 60 minutes a day?

  - 7 days
  - 6 days
  - 5 days
  - 4 days
  - 3 days
  - 2 days
  - 1 day
  - 0 days

46. Do you play sports (this includes any sport in a club or outside a club, but not PE at school)?

  - Yes / No

  If no, why don’t you play sports?

  - I’m not interested
  - I already do enough sports at school
  - Health reasons
  - No time
  - No suitable options nearby
  - Too expensive
  - My parents don’t want me to
  - I don’t have the confidence
  - I don’t know how to start
  - Other: _____________________________

XV. Support Groups

47. Could you imagine that a support group could be something for you?

- Yes / No / I don’t know

48. When you want to inform yourself about topics like health or illness, which options do you use?

- Support groups
- Friends/family/parents
- TikTok, YouTube
- Google
- Social media (Facebook, Instagram)
- Books
- Blogs
- Doctors/medical staff
- Other: ______________________________

XVI. Health-Promoting Activities

49. What sports do you already do outside of school?

- I don’t do sports
- Jogging
- Soccer
- Horseback riding
- Volleyball
- Swimming
- Basketball
- Skating
- Biking
- Martial arts
- Strength/fitness training
- Other: ______________________________

50. What sports would you like to do outside of school?

- I'm not interested in sports
- Horseback riding
- Soccer
- Swimming
- Volleyball
- Basketball
- I already do the sport I want to do
- Other: ____________________________

51. Are there play or sports facilities near your current place of residence that you can easily reach?

- Playground/skater park/soccer field: Yes / No / I don't know
- Sports field/sports hall: Yes / No / I don't know
- Swimming pool: Yes / No / I don't know
- Park, public green spaces: Yes / No / I don't know
- Garden: Yes / No / I don't know
- Other: ____________________________

52. The city of Witten would like to improve the health of children and teenagers. What would you wish for?

(You can write your answer here)

53. Are you interested in the following activities, and would you participate in such activities in the future?

- Sports and exercise activities (e.g., in sports clubs)
  - Yes / No
- Nutrition programs (e.g., cooking classes)
  - Yes / No
- Programs to reduce stress

- Yes / No

- Other (please specify): _______________

  - Yes / No

54. What would motivate you to participate in sports or health activities?

(Multiple answers possible)

- Afternoon programs
- Evening programs
- Weekend programs
- Programs during holidays
- Programs near school
- Programs near home
- Programs for girls only
- Programs for boys only
- School-based programs (e.g., clubs)
- None of the above
- Other (please specify): _______________

55. Which influencers do you follow on social media?

(Multiple answers possible)

- I don’t follow anyone
- I don’t know what that is
- Bibi's Beauty Palace
- Lisa and Lena
- Dagi Bee
- Lena Meyer-Landrut
- Shirin David
- Gronkh
- Heidi Klum
- Mark Forster/Die Lochis

- Capital Bra

- Julien Bam

- Other (please specify): ________________

XVII. Personal Information

56. How religious do you consider yourself?

  - Not at all / A little / Moderately / Quite a bit / Very religious

57. Do you have a religious affiliation?

  - Catholic

  - Evangelical

  - Buddhist

  - Muslim

  - Hindu

  - Free church

  - Jewish

  - Orthodox

  - Esoteric

  - Other: ________________

58. In which country were you born?

- Germany

- In another country, namely: ________________

59. In which country were your parents born?

(Please answer for both parents)

- Mother:

  - Germany

  - In another country, namely: ________________

- Father:

- Germany
- In another country, namely: _______________

60. How would you describe the situation in your family?

(Imagine this ladder represents the structure of society in Germany. At the top are the people with the most money, the highest education, and the best jobs. At the bottom are the people with the least money, lowest education, and worst or no jobs. Now think about your family. On which rung do you think your family would stand? Please check a box next to the ladder.)

© Institute for General Medicine and Outpatient Health Care,

University of Witten/Herdecke, 2021

ID 15

Separate Sheet to be Detached from the Questionnaire

In order to examine the influence of the residential area on your life, we would like to collect your postal code, street, and house number. This is, however, very sensitive information, which we will only use for scientific purposes and in compliance with data protection laws and confidentiality, without making any person identifiable.

You will detach this sheet from the questionnaire. This sheet will be collected separately from the questionnaire and placed in an envelope along with the address sheets of your classmates, which will be sealed in your presence. This sealed envelope will be handed over to the Office for Urban Development, Statistics, and Economic Development of the City of Witten. This will allow us to calculate distances, e.g., to the nearest swimming pool. These distances and the zoning of areas will be sent back to us, together with the identification number on this sheet's top right corner. The identification number allows us to link the information from the Office for Urban Development, Statistics, and Economic Development of the City of Witten to your questionnaire responses without making you identifiable.

This ensures that your sensitive residential address is processed by the Office for Urban Development, Statistics, and Economic Development of the City of Witten without any personal information being known. At the Institute for General Medicine and Outpatient Health Care of the University of Witten/Herdecke, we will not receive your exact address but only distances and general residential area data.

The information about your living environment is crucial for developing health-promoting measures, as it allows us to analyze the strengths and weaknesses of residential areas for young people.

Please write down your postal code, street, and house number below if you are sure you would like to support us with this information:

Street (Please use block letters)

House number

Postal code

City
